# Clinical Rationale of Dual‐Parameter Tumor Markers Reporting Without Reliance on Normal Reference Ranges

**DOI:** 10.1002/jcla.70320

**Published:** 2026-07-27

**Authors:** Jurate Civinskaite, Daiva Gudaviciene, Gintaras Zaleskis, Aleksandras Petrauskas, Dainius Characiejus, Tatjana Ivaskiene

**Affiliations:** ^1^ State Research Institute Centre for Innovative Medicine Vilnius Lithuania; ^2^ Center of Reconstructive Surgery Vilnius University Hospital Santaros Klinikos Vilnius Lithuania; ^3^ Hematology, Oncology, and Transfusion Medicine Center Vilnius University Hospital Santaros Klinikos Vilnius Lithuania; ^4^ Medical Faculty of Vilnius University Vilnius Lithuania

## Abstract

**Introduction:**

This review offers clinicians a novel view of tumor markers (TM). We suggest reporting the TM with two values: the TM level itself and the specific growth rate (SGR). Clinicians are well informed about the limitations of TM‐based cancer screening and early recurrence detection.

**Review of Current TM Interpretation:**

Current TM interpretation is based merely on the TM value. TM cannot provide the information equivalent to histological data. An asymptomatic patient with rising TM is assigned to imaging procedures only if TM is found above the norm.

**Proposal to Report TM in Two‐Parameter Format:**

We offer to universally report TM as a two‐parameter value: (a) TM itself; (b) specific growth rate of TM. The rate of change of TM, rather than the TM norm, is a reliable criterion for recurrence monitoring. For the moment, serial TM values of individual patients are becoming universally available in electronic format.

**Review of Literature Exploring Velocity, Doubling Time, and SGR in Laboratory Reports and Radiological Assessment:**

We offer medical laboratories to put into service a specific growth rate (SGR) parameter which precisely associates: (a) the current TM value; (b) the previous TM value; and (c) the time interval between. Radiologic tumor progression with SGR calculations significantly aided prognosis assessment, but the implementation of SGR in laboratory practice has not been tested. This review focuses on clinical data in which SGR could have been employed to guide therapy or to monitor for recurrence. The obvious advantages of the SGR‐TM format are ease of interpretation (it indicates the percentage change in TM per day), cost‐effectiveness, and mathematical consistency.

## Graphical abstract

We recommend the current practice of tumor marker reporting to upgrade to two‐figure format to include a specific growth rate in parentheses. This can be of great clinical importance, especially in the subnormal range.

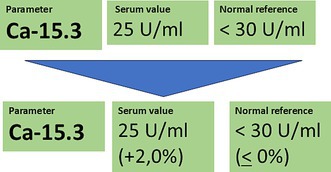

## 1 Introduction

This review offers clinicians a novel interpretation of tumor markers. We recommend reporting them in a standardized format that includes two numeric values: (a) the tumor marker itself and (b) its growth rate expressed as a specific growth rate (SGR). Serum tumor markers (TM) with 100% specificity (undetectable in healthy individuals) and 100% sensitivity (positive at all stages of malignant disease) do not exist. Although TM is usually used as a screening test for cancer detection, once a particular tumor diagnosis has been made, TM may be used to monitor treatment efficacy. Rising TM values within the normal range in the posttreatment setting can be concerning, but exact guidelines for a surveillance strategy in this setting are absent. Also, there is no precise measurable indicator of the TM rate of change (TM‐RoC). Recommendations for initiating clinical and radiological examinations in patients in remission are based on the criterion that TM attains or exceeds the normal cutoff value. The only kinetic TM parameter recommended by ASCO for diagnosis of prostate cancer recurrence/metastasis is a PSA doubling time (DT) shorter than 10–12 months [1]. PSA‐DT is calculated by the clinician, who must carefully retrieve several prior PSA test results and the time between measurements. This manual calculation is time‐consuming, error‐prone, and does not provide confidence.

The early detection of aggressive forms of cancer recurrence in patients without cancer‐related symptoms may offer early salvage treatment opportunities and improved survival. In contrast, symptomatic patients usually have a higher tumor burden and higher TM levels. These higher TM levels often exceed the normal TM cutoff, and patients are referred for radiological investigation at a relatively late stage. Conversely, asymptomatic patients with a significant TM‐RoC below the norm might be unattended. However, a significant shift in TM within the normal range can be detectable when an appropriate TM‐RoC calculation is applied.

We hypothesize that universally available electronic data storage provides clinicians with not only an ordered TM measurement but also a RoC parameter attached to every TM report. There are only two options to define RoC by this approach, assuming a minimal residual tumor regrowth pattern is exponential: (a) a DT; (b) a specific growth rate (SGR). A third option—velocity—is not appropriate since it ignores the presence of an exponential regrowth pattern.

### 1.1 Tumor Marker Norm—An Anchoring Bias

Serum TM cannot provide information equivalent to that obtained from histological examination. Clinicians often compare TM values to the “norm,” which is intended to represent a fixed cutoff for a healthy population. This approach applies to all available laboratory test interpretations, including WBC, CRP, D‐dimer, and Troponin. However, the direct association between TM presence in serum and the presence of a specific tumor is somewhat simplistic. TM is not histology data equivalent to what is desired. Decision‐making based on the comparison of TM to the normal range of a healthy population would be possible only in the case of its 100% specificity and 100% sensitivity. Unlike WBC, CRP, Troponin, or other conventional tests, TM tends to retain its abnormal values for months and years. The change in TM value over time for each patient is far more important than the “norm” of a healthy population. This particularity of TM led to the assertion of certain principles of “sensitive use of TM” by P.Stieber [2]: (a) the principal application of all TM is to monitor progress and response after surgery and/or radiotherapy, chemotherapy; (b) TM kinetics, not individual values, are critical in this respect; (c) changes in the concentration of tumor markers—independent of reference ranges—are clinically relevant and are detectable up to 26 months ahead of the diagnosis of recurrence; (d) the most frequent false interpretation of TM is based on the overestimation of normal cutoff values and underestimation of the individual baseline values; (e) only the individual kinetics are of relevance. After the first course of therapy, intended to be curative, each of these levels should be considered as its specific new “reference level”. It serves as the baseline level for further monitoring; (f) in the post‐treatment surveillance period, the upper reference limit of healthy subjects is no longer of significance, and instead, the kinetic changes in every single patient are of particular importance.

Every lab test is provided with “norm” reference values. For instance, PSA measurement data is also pinned with a 0–4 ng/mL norm range next to the measurement result. However, several years following prostatectomy, the upper PSA reference limit of healthy subjects (4 ng/mL) is no longer relevant. Relentless attempts to compare TM value to the norm resemble a typical behavioral phenomenon known as an anchoring bias. The most worrying “normal range anchoring” was reported in the study of men monitored for recurrence of prostate cancer [3]. PSA levels should become *undetectable* following tumor removal [4]. However, HCPs tend to habitually use a PSA threshold that is too high (4.0 ng/mL) to monitor patients postoperatively [3]. This study investigated whether HPCs might be mistakenly using “normal” *screening* cutoffs (4 ng/mL) as their primary threshold for doing aggressive cancer surveillance in the *post‐prostatectomy* setting. There must have been a lot of missed opportunities while referring to the PSA norm in these patients. Furthermore, this study found that postsurgical guideline concordance sharply increased when PSA values exceeded the value of 4 ng/mL, suggesting HCPs were using a *screening* threshold to make decisions about cancer *recurrence*. Of course, the screening threshold of 4 ng/mL is not relevant and dangerously high in the post‐prostatectomy setting. The PSA value to be considered in the postoperative setting is 0.2 ng/mL (biochemical recurrence). Additional delays occur when a transition from 0 to 0.2 ng/mL is not spotted by HCP. It may take years to accomplish a transition of care from HCPs to oncologists. The risk of delayed referrals and missed opportunities might be linked to the PSA norm indicated in laboratory reports. A “norm anchoring” was also found in questionnaire data on HCP familiarization with “undetectable PSA” requirements in the postoperative setting [5]. Just 13% of questioned HCPs provided correct answers. Other participants mentioned worrisome high PSA cutoff values: 10 ng/mL, 4 ng/mL, or 1 ng/mL.

We would like to recommend the implementation of an additional parameter that should be reported alongside every TM result. The proposed indicator is the specific growth rate (SGR). Attaching the SGR value and its norm (SGR ≤ 0%/day) to every TM report must help avoid trivial interpretation mistakes. The SGR provides valuable information in case TM movement occurs in the normal or abnormal range. The SGR was used by radiologists performing MRI, sonography, or CT scans to evaluate the rate of tumor progression [6, 7, 8, 9, 10]. For the calculation of SGR, the equation to be used is SGR = ln(2) (V2/V1)/(t2‐t1) [11]. Once the V2 (the current TM value) is higher than V1 (the former TM), the SGR numbers are positive (+), indicating TM increase over a period. In contrast, SGR values will be negative (−) in case the current TM (V2) value is lower than the former one (V1). The time difference (t2‐t1) between the two measurements must be calculated in days.

**FIGURE 1:**
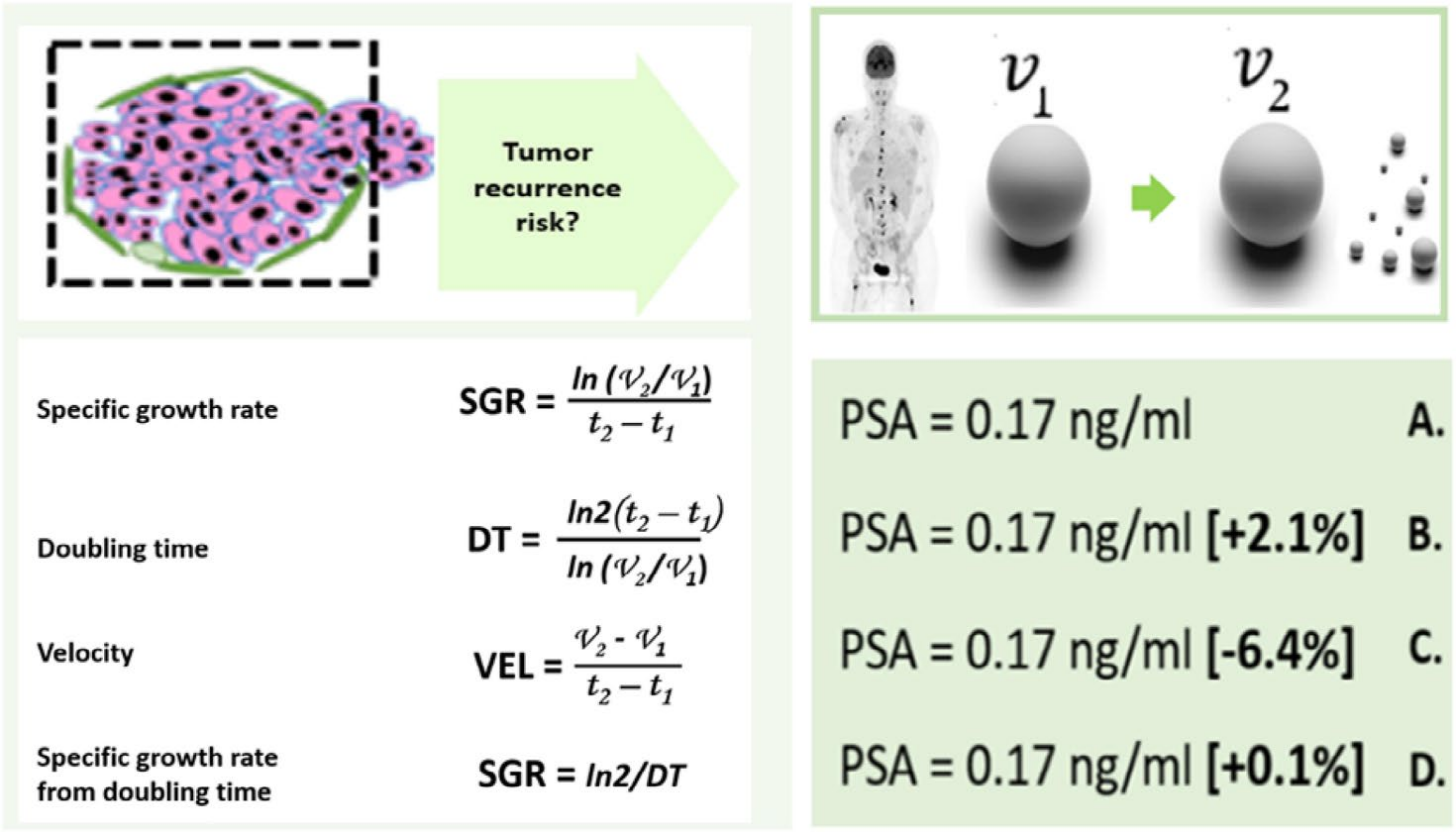
Practical equation to calculate SGR and related parameters. The current TM value (V2), former TM value (V1), and exact time interval in days (t2–t1) are used. The recurrence risk evaluation in post‐treatment follow‐up can be done by TM doubling time and velocity calculation. Velocity is not as accurate as SGR. DT is as perfect as SGR for logarithmic approximation, but DT is indefinite if the tumor is not growing. Laboratory can supply the clinician with TM SGR values if the former measurement is available. Examples of PSA interpretation when the test result is pinned with SGR data (B–D).

An example of such a TM‐SGR‐pinned report is shown in Figure 1A–D. Currently, a clinician is provided with a PSA (or other TM) value that indicates just the concentration of a biomarker, for instance, 0.17 ng/mL (A). The value in this example is rather close to the PSA biochemical recurrence criterion (0.2 ng/mL). Our suggestion is to attach an SGR value to this PSA report, indicating the percent of change per day. For instance, the PSA value is supplied with SGR indication in the following format: 0.17 ng/mL [+2.1%/day] (Figure 1B). It indicates that PSA is increasing by 2.1% per day since the last PSA measurement. A seemingly low percentage increase provided in the Figure 1B example should not be misleading. In fact, this example is a red flag specifying that the threshold of biochemical tumor recurrence (0.2 ng/mL) will be reached in 8.1 days. This patient cannot be appointed for PSA testing according to his surveillance schedule since biochemical recurrence will occur earlier. Instead, a confirmatory PSA test is recommended right now. Conversely, the Figure 1C example indicates that the 0.17 ng/mL value is dramatically declining. This is a positive finding if PSA is tested 1 month after radical prostatectomy. The slower postoperative decline, for instance, SGR = −1.0%/day, might probably indicate high postoperative margins left or occult metastases. Finally, the example in Figure 1D indicates the presence of a tumor in remission. Although the “red flag” value of biochemical recurrence (0.2 ng/mL) is quite close, no immediate action (as is the case in Figure 1B) is needed. The clinician should not be involved in the “homework” of the calculation of SGR; this can be accomplished by the lab.

The question is what numerical values of SGR‐TM can indicate tumor aggressiveness. The guidelines for every TM and specific tumor type are yet to be established since TM‐SGR has never been tested in clinical studies. One of the objectives of our paper is to recommend that SGR be included in every TM report. Data on TM‐SGR interpretation can be obtained from investigations reporting radiological SGR numerical values in progressing tumors with variable aggressiveness. Dejaco D. and coworkers [6] classified head and neck squamous cell carcinoma according to SGR values into three groups: (a) less than 0.3%/day; (b) 0.3%/day ≤ SGR < 3.0%/day, and (c) more than 3.0%/day. A dramatic difference in overall survival was associated with SGR values. The shortest overall survival was observed in the fastest‐growing tumors (SGR ≥ 3.0%/day). Another hint for the interpretation of the TM‐SGR value can be obtained from the guidelines recommending the decision‐making based on the TM‐DT criterion. For instance, Huang with coauthors [12] recommends that active observation following radical prostatectomy may be referred to a PSA‐DT cutoff of 12 months for patients not exhibiting signs of biochemical recurrence (0.2 ng/mL). The DT and SGR are interrelated: SGR = ln(2)/DT(11). Thus, 12‐month DT (365 days) can be expressed as SGR = +0.19%/day. So, patients revealing SGR less than +0.19%/day values according to the proposed DT criteria are at low risk of biochemical recurrence. There are several advantages to using SGR rather than DT [11, 13]. When the TM value does not change over time (stable disease), the denominator in the classical DT equation is zero. This makes DT value indefinite, whereas SGR under the same conditions indicates the absence of TM progression (SGR = 0%/day). Clinicians preferring DT format in double‐parameter data reporting should be informed that the symbol ∞ means stable tumor. Mathematical advantages of SGR were demonstrated via Monte‐Carlo simulation and modeling (positive skew and asymmetrical distribution of DT when testing on clinical data). This led to recommendations to explore SGR indicators not only in tumor radiological volume progression measurements but also in serial TM evaluation [13]. However, we are not aware of any TM‐SGR study published. We expect that TM reports presented to clinicians with the SGR value will be easily accepted. This should immensely simplify clinicians' daily routines, automatically attaching the current TM value to the previous value and to the time interval between these two measurements.

### 1.2 An Option of the TM Lab Report Supplied With SGR

**FIGURE 2:**
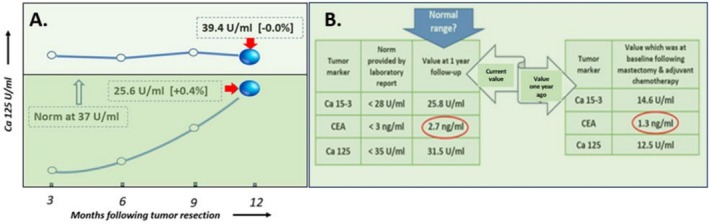
The Importance of TM alteration in the subnormal range. (A) Comparing TM value to the cut‐off level of healthy individuals (“norm”) might be misleading if it does not consider previous TM findings. An example of two Ca125 measurements that are interpreted in comparison to the norm. The standard approach ignores the subnormal (25.6 U/mL) case. The addition of SGR to each of these measurements indicates that a subnormal TM (25.6 U/mL) is exhibiting significantly more aggressive behavior with the potential to exceed the norm within 92 days. (B) A strategy proposed by Di Gioia D. and coauthors [16] to detect breast cancer recurrence based on individual TM change values. This figure is made referring to the proposal of authors [16] to detect recurrence once CEA is increasing > 100%, regardless of the normal range or not.

The standard approach for post‐treatment monitoring is a physical exam and a review of symptoms, anywhere from every three to 6 months for the first 2–3 years, then every 6 months until year five, and annually thereafter. So, TM tests are also done at the same time points. In fact, any doubtful result at any time point can be repeated and downloaded into the SGR calculation system with an additional SGR value obtained. The SGR can be calculated, including more than two TM measurements. A KELIM parameter, which is somewhat similar to a negative SGR, is calculated with the help of three TM determinations strictly within 100 days [14, 15]. The SGR can be calculated with any time interval and an unlimited number of TM determinations. In case there was no additional treatment during the TM testing interval; then, the SGR should remain unchanged. Thus, a threatening relapse (positive high SGR) might become visible while TM is still in a normal range. A characteristic example is presented in Figure 2A. An asymptomatic patient in the post‐ovarian tumor resection period exhibiting normal Ca125 levels (25.6 U/mL) will be considered a patient in remission, regardless of high SGR in the normal range. This example demonstrates the necessity of double‐figure TM reporting, which must include SGR. The high Ca125 levels exceeding the norm will occur in about 3 months for this patient.

The TM exceeding norm has been shown to detect 40%–60% of breast cancer recurrences before clinical or radiological evidence of disease, with a lead time between 2 and 18 months [16, 17, 18, 19]. However, the exact guidelines for decision‐making in the case of a TM‐only rise remain debatable. Nicolini and coauthors [19] compared the survival between TM‐guided salvage treatment and those treated after radiological confirmation of disease recurrence following standard guidelines. Importantly, TM‐only guided salvage treatment significantly prolonged the disease‐free and overall survival. An interesting approach to the solution of the dilemma of TM rise was suggested by di Gioia and coauthors [16]. After primary therapy, patients underwent tumor marker monitoring for CEA, Ca 15–3, and Ca 125 at intensified 6‐week intervals, exploring innovative diagnostic aftercare algorithms. A reproducible, previously defined increase (CEA by 100%; Ca 15–3 by 75%; Ca 125 by 150%) of single or combined markers compared with the baseline value was considered a strong indicator of recurrent disease. An example of this approach is shown in Figure 2B, where all three markers are matching these increase criteria, but are not easy to spot since all three TMs are within the normal range (although CEA increase equals 107%, Ca 15–3 to 77% and Ca125 to 152% in our example). The clinician tends to interpret CEA rise as insignificant, “by just 1.4 ng/ml”. Importantly, the percentage, not an absolute value, can indicate significant TM change. This approach [16] resulted in early relapse detection in 65.9% of patients, as well as a high 13.6% rate of secondary malignancy presence. Notably, secondary malignancies detected in this study were treated at an early stage. We would recommend the data shown in Figure 2B to be presented as follows: Ca 15–3 = 25.8 U/mL [+0.16%/day]; CEA = 2.7 ng/mL [+0.20%/day]; Ca 125 = 31.5 U/mL [+0.26%/day]. These SGR numerical values might be adopted as a recurrence risk criterion since similar SGR criteria appeared to be predicting progression risk in CT, ultrasound, or MRI examination studies [6, 7, 8, 9, 10].

The norm anchoring can often be seen in cases where a modified TM norm is being adopted for the specific clinical situation. For instance, CEA was suggested to exhibit better sensitivity and specificity if different norms were considered for different tumors [20]. Usually, CEA norm values provided with reagent inserts claim a 2.5 ng/mL cut‐off for nonsmokers and 5.0 ng/mL for smokers. Surprisingly variable CEA cut‐off levels of control norm differing from 2.5 ng/mL were proposed in different tumor types [21, 22, 23, 24, 25, 26, 27, 28]. So the norm pinned with SGR will definitely solve this inconsistency when CEA norm is found so much different in various tumors.

Nicolson BD and coworkers [29] performed a review and meta‐analysis of 52 studies, suggesting an optimal CEA value for the detection of recurrent colorectal cancer. Seven studies from this review offered 2.5 ng/mL; however, the other 23 studies proposed a 5.0 ng/mL cut‐off level, and another seven studies advocated 10 ng/mL. This meta‐analysis report [29] concluded that the optimal level should be 10 ng/mL. The normal range and organ specificity of different TM (e.g., PSA specificity for prostate) are rather relative definitions. For instance, PSA norms for men and for women are set at dramatically different levels. The “normal” serum PSA concentration in females is approximately 1000‐fold less than that in males (0–0.004 ng/mL) [30]. However, PSA can serve as an excellent TM in women's breast cancer diagnosis and treatment monitoring if serial measurements are done [30, 31, 32]. The percentage of breast cancer patients with free PSA as the predominant molecular form in serum was five times higher than that of healthy women [33]. Also, PSA decreased in the serum of breast cancer patients after surgery, and free PSA showed a high diagnostic specificity (approximately 96%) in comparison to women free of breast cancer or with benign breast disease. Attaching SGR to every TM report does not require any norm. The SGR must help to compare the current TM to the previous one. And to indicate a rate of change for that patient.

### 1.3 Why Should SGR Be Preferred Over TM Velocity?

Doubling time (DT) as well as SGR is a correct approximation that assumes tumor growth is not linear but exponential. However, DT was rarely used as a criterion of TM increase in the normal range. Instead, linear thinking in a nonlinear world was explored most. De Langhe and coauthors [34] associate this phenomenon with psychological linearity preference. A sudden relapse of a tumor that was silent for years is not a rare occurrence if linear tumor progression is anticipated. Instead, exponential growth must always be considered. The exponential model points out that a minuscule tumor possesses the same SGR (or the same DT) as a large tumor does. A tumor size doubling from 1 mm in diameter will remain undetectable, whereas the doubling of a 3 cm tumor will look like a “sudden recurrence”. So, the expectation of long‐standing velocity as opposed to steady SGR or DT will lead to a completely different interpretation. The clinical application of velocity was popular in screening prostate cancer by serial PSA determinations in healthy men. This approach is still advocated, and patients or physicians can check their PSA velocity values by inserting their PSA data into calculators available on the web [35]. Unsurprisingly, PSA velocity appeared to be of little use in prostate cancer detection in men with prior negative biopsies [36].

An application of postoperative Ca19‐9 velocity to evaluate prognosis in patients with pancreatic cancer was also attempted [37]. The authors hypothesized that postoperative Ca19‐9 velocity was likely related to relapsed tumor growth. The Ca 19–9 velocity predicted disease‐free and overall survival and predicted the latter with better accuracy than did baseline Ca19‐9 norm levels. A numerical Ca19‐9 velocity cut‐off that predicted imminent radiological recurrence (95 U/mL/4 weeks) was also offered in this study. The authors recommended determining Ca19‐9 levels at monthly intervals. It would be of interest to carry out the same study using SGR instead of VEL. Interesting data were reported by Hing JX [27] and coworkers on the VEL criteria implementation for the detection of recurrence in breast cancer patients with the help of CEA and Ca15‐3 serial measurements. The optimal cut‐off values for Ca 15–3 and CEA velocity were derived to be 2.5 U/mL/year and 1.2 ng/mL/year, respectively. ROC data of velocity of both TM were superior to the other known parameters, for example, lymph node involvement, clinical stage, and NPI scores. Raised TM prompted further cross‐sectional imaging and detected recurrences with an average lead time of 4 months. The cut‐off values for Ca 15–3 and CEA levels at the time of recurrence were referred to as 24.8 U/mL and 4.3 ng/mL, respectively. Thus, it was a standard norm again. Potentially linear VEL simplification did not add to the norm criteria in this setting. We hypothesize that the DT or SGR would have been more precise in signaling recurrence at an early stage of subnormal TM levels than at TM norm. The difference is that VEL calculates (V2 − V1)/*t*, whereas SGR estimates essentially the same TM change over time but in logarithms: ln(V2)−ln(V1)/t.

Experimental and human tumors were demonstrated to grow following the Gompertzian pattern, which at an early stage (supposedly still having subnormal TM) is exponential [38, 39, 40, 41]. It is based on studies fitting clinical data for the survival of untreated breast cancer patients, for the progression of radiological tumor volumes on serial paired mammograms, for time‐to‐relapse following mastectomy and for immunoglobulin secretion by myeloma.

**FIGURE 3:**
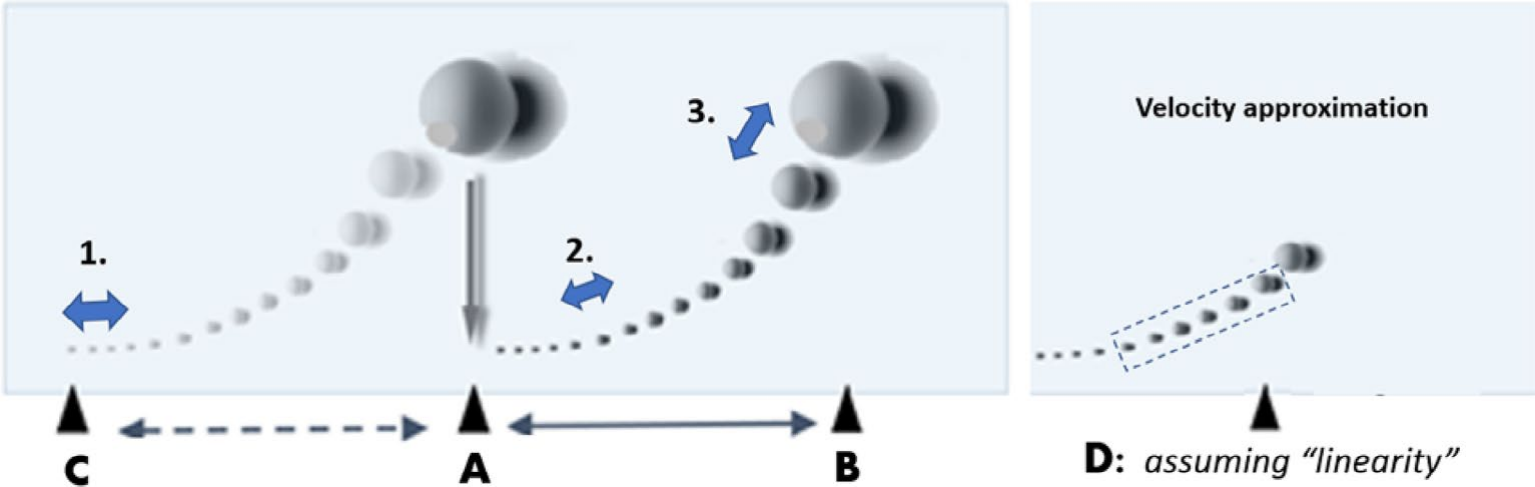
Exponential vs. a linear expectation of TM progression and Callin's law. Following tumor excision (A) the exponential regrowth pattern continues in the remission period (interval A–B). According to Callin's law [38], these two segments (C–A and A–B) are identical. It might seem that the recurrent tumor (interval increment [3] “suddenly” relapsed (e.g., increased in size from 1 cm to 10 cm)). However, tumor size increases from 0.1 mm to 1.0 mm (interval [1] or [2] is equally the same (10‐fold)). The clinician is presented with a TM and radiological findings, observing incremental change. A small increment (0.1 cm) is not detectable by radiological examination and might create an illusion of linear growth when examined radiologically in certain increments at later stages (D).

The attempts to ascribe a linear pattern (velocity) to TM increase are currently in the physician's intuitive daily use. For instance, the clinician is presented with an ovarian cancer case, 5 years after cytoreductive surgery and adjuvant chemotherapy with Ca125 = 120 U/mL. Three months ago, the same patient exhibited Ca125 = 80 U/mL; the velocity is 40 U/mL/90 days. This patient will be appointed to the radiological and clinical examination just because both values are above the norm (35 U/mL), not by velocity or other dynamic TM evidence. From the point of view of correct interpretation, this Ca125 shift from 80 U/mL to 120 U/mL is identical to the shift from 8 U/mL to 12 U/mL. The latter shift will probably remain unnoticed because it is taking place in the subnormal range. The SGR of both is +0.45%/day, which is rather high, and the patient with this SGR within the Ca125 norm should be considered for appropriate examination. Exponential growth assumes that the tumor is growing at the same rate years before GRThis slow post‐treatment tumor progression is known as Collins' law. Collins' law states that the period of risk for tumor recurrence is the age of the child at diagnosis plus 9 months [42]. For adult breast cancer, the median time of local recurrence was 2.7 years, whereas for lung and colorectal cancer, it was 1.5 years. Based on Collins' law calculations, the age of these solid tumors was found to be 3 to 6 years [43]. Nevertheless, a small percentage of pediatric malignancies recur suddenly [44] as if the tumor in the remission period was silent for a prolonged period and then suddenly relapsed. The mismatch between clinical intuition (which usually anticipates linear progression) and large‐mass tumor relapse is shown in Figure 3. Once the SGR approximation is applied to the TM analysis, then the unexpectedly large tumor mass relapse shouldn't be a surprise. The tumor mass increase is the same at very small tumor mass (intervals “1” and “2”, Figure 3) as well as larger (interval “3”, Figure 3). Intuitively, clinicians tend to interpret an exponentially growing tumor in a linear pattern (Figure 3D). Thus, the clinical application of SGR or DT criteria is of great value, especially if TM is fluctuating in the normal range. Two‐parameter reporting can be of great help if TM is still in a subnormal range. This “hockey stick” graphical pattern of sudden TM increase was demonstrated by Andersson and coauthors [45] performing serial measurements of TM (including Ca 125) in healthy women for 18 consecutive years before ovarian cancer diagnosis. The study revealed that roughly 3 years before clinical cancer manifestation, TM starts rising, potentially signaling the presence of malignant tissue. This study was possible since frozen serum samples of 18,314 women participating in the CARET trial were stored for 18 years. In fact, elevated preclinical Ca125 value years before ovarian cancer diagnosis was reported in several studies [46, 47, 48, 49, 50]. The Ca‐125 levels from these screening trials for ovarian cancer have indicated that serial Ca‐125 levels may identify cases better than a fixed Ca‐125 cutoff norm. Normal range SGR criteria were not adopted for ovarian cancer screening. Ovarian cancer screening observations also revealed a so‐called Ca‐125 jump‐up pattern several years before diagnosis [46]. Therefore, the exponential approximation of TM shift in asymptomatic disease is quite promising. This makes SGR calculation a reliable tool for screening and minimal residual disease monitoring.

### 1.4 Early Rise of TM SGR Might Also be Associated With Non‐Oncological Disease Progression

All SGR calculations must be determined by exploring the same laboratory technology, preferably also at the same medical facility. Potentially better value of assessing TM dynamics (especially in the low subnormal range) would be if multiple serial TM values were assessed in the same laboratory. The addition of SGR to TM value should catch attention earlier and might detect very small but diagnostically important movements of TM. There are, however, some traps unrelated to malignant disease that can also become “more visible” when SGR is included in the TM report. For instance, Ca125 appeared to be an excellent marker for monitoring congenital heart failure [51, 52, 53, 54] and early carotid atherosclerosis [55]. These pathologies can also exhibit slow progression and become visible by SGR earlier than by TM's normal cutoff. The authors of the study [56] measured six tumor biomarkers (AFP, CA125, CA15‐3, CA19‐9, CEA, and CYFRA 21‐1) and detected their high predictive value for cardiovascular disease and mortality in the *general population*. The implementation of SGR in this scenario must consider the probability of cardiovascular disease in cancer patients. The cardiotoxicity of chemotherapy must also be considered in serial TM measurements.

Implementing SGR in the form of every single TM test (+/‐SGR) does not change any currently available guidelines or treatment strategies based on established TM norm criteria. Putting SGR into practice provides clinicians with supplemental information. The perception of exponential TM growth is most convenient when presented as an SGR value. It is easy to understand and interpret. The calculation is up to the lab and must be arranged via automatic equation installation in daily practice. The SGR norm [≤ 0%/day] is easily understandable. The SGR‐TM should be a useful tool in research and especially in practice. While double‐parameter reporting seems plausible, it is not yet clinically validated. We just want to note that clinical trials in this direction seem quite attractive. We propose implementing SGR into serial TM measurement practice as an algorithm that enhances TM data interpretation. It is a simple, cost‐effective, and mathematically robust.

## Author Contributions

J.C.: conceptualization; D.G.: methodology; G.Z.: writing – original draft, visualization; A.P.: literature search; DC: writing – review and editing; T.I.: supervision and project administration.

## Funding

This project was funded by the Research Council of Lithuania (LMTLT), agreement No. S‐MIP‐22‐4.

## Conflicts of Interest

The authors declare no conflicts of interest.

## Data Availability

Data availability claim is not applicable to this article as no datasets were generated or analysed during this study. This is a theoretical review paper.
